# RACK1 mediates NLRP3 inflammasome activation during *Pasteurella multocida* infection

**DOI:** 10.1186/s13567-023-01195-5

**Published:** 2023-09-08

**Authors:** Jinrong Ran, Hang Yin, Yating Xu, Yu Wang, Gang Li, Xingping Wu, Lianci Peng, Yuanyi Peng, Rendong Fang

**Affiliations:** Joint International Research Laboratory of Animal Health and Animal Food Safety, College of Veterinary Medicine, Southwest University, Chongqing, 400715 China

**Keywords:** *Pasteurella multocida*, NLRP3 inflammasome, RACK1, NLRP3, NF-κB

## Abstract

*Pasteurella multocida* is a gram-negative bacterium that causes serious diseases in a wide range of animal species. Inflammasomes are intracellular multimolecular protein complexes that play a critical role in host defence against microbial infection. Our previous study showed that bovine *P. multocida* type A (PmCQ2) infection induces NLRP3 inflammasome activation. However, the exact mechanism underlying PmCQ2-induced NLRP3 inflammasome activation is not clear. Here, we show that NLRP3 inflammasome activation is positively regulated by a scaffold protein called receptor for activated C kinase 1 (RACK1). This study shows that RACK1 expression was downregulated by PmCQ2 infection in primary mouse peritoneal macrophages and mouse tissues, and overexpression of RACK1 prevented PmCQ2-induced cell death and reduced the numbers of adherent and invasive PmCQ2, indicating a modulatory role of RACK1 in the cell death that is induced by *P. multocida* infection. Next, RACK1 knockdown by siRNA significantly attenuated PmCQ2-induced NLRP3 inflammasome activation, which was accompanied by a reduction in the protein expression of interleukin (IL)-1β, pro-IL-1β, caspase-1 and NLRP3 as well as the formation of ASC specks, while RACK1 overexpression by pcDNA3.1-RACK1 plasmid transfection significantly promoted PmCQ2-induced NLRP3 inflammasome activation; these results showed that RACK1 is essential for NLRP3 inflammasome activation. Furthermore, RACK1 knockdown decreased PmCQ2-induced NF-κB activation, but RACK1 overexpression had the opposite effect. In addition, the immunofluorescence staining and immunoprecipitation results showed that RACK1 colocalized with NLRP3 and that NEK7 and interacted with these proteins. However, inhibition of potassium efflux significantly attenuated the RACK1-NLRP3-NEK7 interaction. Our study demonstrated that RACK1 plays an important role in promoting NLRP3 inflammasome activation by regulating NF-κB and promoting NLRP3 inflammasome assembly.

## Introduction

*Pasteurella multocida* (*P. multocida*) is an opportunistic pathogen that causes infectious diseases in the respiratory tracts of domestic and wild animals as well as humans [1], and it causes diseases including bovine respiratory disease [2], haemorrhagic septicemia [3], and fowl cholera [4]. Currently, *P. multocida* is classified into five serogroups, including A, B, D, E and F [5]. According to the capsule, serotype A of *P. multocida* is the major pathogen that causes pneumonia and bovine respiratory disease complex (BRDC), resulting in substantial economic losses in the bovine industry [6]. To date, there are neither efficient vaccination strategies nor antibiotics to prevent and treat *P. multocida* infection.

The innate immune system is the first line of host defence against exogenous microbial invasion and endogenous damage signals. The NLRP3 inflammasome is a cytoplasmic multiprotein complex that is an important component of the innate immune system, and its activation plays a critical role in the host response to pathogens [7]. It is well known that NLRP3 inflammasome assembly and activation lead to the secretion of proinflammatory cytokines, including IL-1β and IL-18. It has been reported that NLRP3 is activated by different pathogens, such as *Mycobacterium tuberculosis (M.tb)* [8], *Staphylococcus aureus* [9], and *Clostridium septicum* [10], and NLRP3 plays an important role in protecting the host against infection. Similarly, our previous study showed that *P. multocida* activates the NLRP3 inflammasome, leading to caspase-1 activation and subsequent IL-1β secretion in vivo and in vitro, and this NLRP3 inflammasome activation depends on macrophage phagocytosis [11]. Furthermore, *P. multocida*-induced NLRP3 inflammasome activation is mediated by the efflux of potassium ions (K^+^) and NIMA-related kinase 7 (NEK7) [12]. However, NLRP3 activation is thought to be involved in multiple upstream signals, such as chloride efflux [13], defective mitochondrial function [14], and Ca^2+^ signalling [15]. Whether other upstream signals modulate *P. multocida*-induced NLRP3 inflammasome activation is still unknown [16].

RACK1 is a highly conserved tryptophan-aspartate repeat (WD-repeat) protein and is currently recognized as a multifunctional scaffold protein that is involved in a wide range of biological processes, such as viral infection [17], cell migration [18], and apoptosis [19]. It has been reported that RACK1 participates in NLRP3 inflammasome activation by promoting NLRP3 conformational changes and inflammasome assembly [20]. For example, *M.tb* EST12 increases resistance to *M.tb* infection in vivo and in vitro by binding to RACK1, inducing NLRP3-ASC complex formation and activating the NLRP3–caspase-1–GSDMD–IL-1β pathway [21]. However, the role of RACK1 in the development of inflammation induced by *P. multocida* infection is still unknown.

In this study, we investigated the mechanism by which RACK1 mediates *P. multocida*-induced NLRP3 inflammasome activation. The results showed that RACK1 protein levels decreased during *P. multocida* infection. In addition, the results showed that RACK1 protein expression in different tissues of mice and primary macrophages was markedly decreased during *P. multocida* infection. Furthermore, overexpression of RACK1 promoted NLRP3 inflammasome activation by activating the NF-κB signalling pathway during *P. multocida* infection. In addition, *P. multocida* induced the interaction of RACK1 with the NLRP3 protein to promote NLRP3 inflammasome assembly, leading to the activation of caspase-1, which promotes the maturation and secretion of cytokines, including IL-1β and IL-18. Our study demonstrated the regulatory role of RACK1 in NLRP3 inflammasome activation during *P. multocida* infection.

## Materials and methods

### Mice

Wild-type (WT) C57BL/6 mice were purchased from Chongqing Academy of Chinese Material Medical (Chongqing, China). All the mice were maintained under specific pathogen-free (SPF) conditions and used at the age of 8–10 weeks old. All the animal experiments were approved by the Institutional Animal Care and Use Committee (IACUC) of Southwest University, Chongqing, China (IACUC-20221022-03).

### Bacterial strains and culture conditions

The highly virulent bovine *P. multocida* capsular type A PmCQ2 (GenBank Accession number: LIUN00000000) was isolated from the lungs of calves with pneumonia in Chongqing, China [22] and stored at − 80 ℃. The bacteria were incubated on Martin’s agar plates at 37 ℃ for 18–24 h. Then, a single colony was incubated in 5 mL Martin Broth (Solarbio, China) supplemented with 5% FCS (Gibco, USA) at 37 ℃ for 12 h. The bacterial concentrations were determined by colony counting assay, and the bacteria were diluted with cell culture medium to the indicated concentrations for experimental use.

### *Pasteurella multocida* infection in primary mouse peritoneal macrophages

Primary peritoneal macrophages (PECs) were collected as previously reported [11]. Briefly, mice were intraperitoneally injected with 2–3 mL 4% thioglycolate medium (Eiken, Tokyo, Japan). After 3–4 days, the mice were anaesthetized with ether, and PECs were collected by peritoneal lavage. Then, the PECs were suspended in RPMI 1640 medium supplemented with 10% FCS or Opti-MEM (Gibco, USA). The cells were seeded at 1 × 10^6^ cells/well in 12-well (or 6-well) plates or 2 × 10^5^ cells/well in 48-well plates. These cells were incubated in a humidified 37 ℃ incubator with 5% CO_2_. After 2 h of incubation, the nonadherent cells were removed, and the adherent cells were infected with PmCQ2 at a multiplicity of infection (MOI) of 1 for 9 h. Then, 100 µg/mL ciprofloxacin (Solarbio, China) was added for an additional 15 h. After 24 h of incubation, the supernatants and cell lysates were collected and used in the assays described below.

### *Pasteurella multocida* infection in vivo

WT mice were anaesthetized by intraperitoneal (i.p.) injection with 1.5% pentobarbital at a dose of 33.5 mg/kg (MREDA, Beijing, China), and then the mice were intranasally infected with 1000 CFU PmCQ2 or PBS as a control. After 48 h of infection, all the mice were euthanized by ethyl ether, and then the heart, spleen, liver, lung, and kidney were collected and homogenized with PBS for use in the assays described below.

### Overexpression of RACK1 in cells

RACK1 cDNA was reverse transcribed from the total RNA of PECs and amplified using primers (RACK1- F: 5′-cccaagcttatgaccgagcagatgacc-3′ and RACK1-R: 5′-cggaattcttagcgggtaccaatagtta-3′, Sangon Biotech) that were designed according to the NM_008143.3 sequence. Then, the PCR product was gel purified and cloned into the pMD19-T vector (TaKaRa, Dalian, China), which was transformed into competent DH5α cells. Next, positive clones were picked from LB agar plates and cultured in LB for 12 h. Subsequently, RACK1 fragments in the pMD19-T vector were digested using EcoR I and Hind III and then gel purified. Moreover, the pcDNA3.1(+) vector (Invitrogen, V79020) was also digested using EcoR I and Hind III, and then the expected fragment (5387 bp in length) was gel purified. The two purified target fragments were ligated with T4 ligase at 4 °C overnight. Finally, the products (pcDNA3.1-RACK1) were subsequently transformed into competent DH5α cells, and positive clones were selected for bulk culture and sequencing confirmation.

Cells were cultured in 6-well plates as described above and then transfected with 2.5 µg expression plasmid (pcDNA3.1-RACK1) or empty vector (pcDNA3.1) as well as 3.75 µL Lipofectamine 3000. At 48 h post-transfection, the cells were infected with PmCQ2 at a MOI of 1 for the indicated time, and further experiments were performed as described below.

### Cell viability

Cells were prepared and treated as described above. After incubation, 150 µL of 10% WST reagent was added according to the manufacturer’s protocol. After 20 min of incubation, absorbance was measured at 450 nm with a microplate reader (Bio-Rad, Japan) and corrected according to the absorbance at 630 nm. Untreated cells were used as a negative control. The percent of cell viability (%) was calculated as (OD_450nm_ of sample) / (OD_450nm_ of control) × 100%.

### Adhesion and invasion assays

Cells were prepared and treated as described above. Adherent macrophages were infected with *P. multocida* (MOI = 1) for 3 or 7 h. Then, the macrophages were washed with PBS three times to remove nonadherent bacteria. Subsequently, the cells were lysed using 0.1% Triton X-100 in PBS, and the numbers of adherent bacteria were quantified. For the invasion assay, cells were cultured for an additional 30 min in the presence of 100 µg/mL ciprofloxacin to kill the extracellular bacteria. Finally, cell lysates were diluted with PBS and grown on Martin’s agar plates at 37 ℃ for 18–24 h to determine the number of colony-forming units (CFUs) of adherent and invasive bacteria, respectively.

### Enzyme-linked immunosorbent assay (ELISA)

Cells were prepared in 48-well plates and infected with PmCQ2 as described above. After infection, the supernatants were collected, and the levels of cytokines were determined by enzyme-linked immunosorbent assay (ELISA) according to the manufacturer’s instructions. The kits that were used in this study included IL-1β and TNF-α kits (Invitrogen, CA, USA).

### Western blotting

Cells were prepared in 12-well plates and infected with PmCQ2 as described above. After the indicated time of infection, the supernatants were collected and concentrated using 20% (w/v) trichloroacetate (TCA), and then cells were lysed with 1 × SDS loading buffer (Beyotime, Beijing, China). Next, the supernatants and cell lysates were separated by 10–15% SDS‒PAGE and subsequently transferred to polyvinylidene difluoride (PVDF) membranes by electroblotting. The membranes were blocked with 5% nonfat dry milk and then immunoblotted with the indicated antibodies (Abs) including anti-mouse caspase-1 p20 Ab (AG20B-0042) (AdipoGen, San Diego, USA), goat anti-mouse IL-1β Ab (AF-401-NA) (Bioss, Beijing, China), rabbit anti-mouse ASC Ab (67, 824) (Cell Signaling Technology, Danvers MA, USA), anti-NEK7 Ab (ab133514) (Abcam, Cambridge, UK), and anti-rabbit RACK1 Ab (D59D5) (Cell Signaling Technology, Danvers MA, USA). Finally, distinct protein bands were detected by ECL detection reagent (Biosharp, China). The Western blotting bands were quantified to obtain numerical values using image software.

### Quantitative RT-PCR

Cells were infected with PmCQ2 as described above. After infection, total RNA was extracted from cells using TRIzol Reagent (Life Technologies Carlsbad, CA, USA) according to the manufacturer’s protocols. Complementary DNA was generated using the PrimeScript® RT Reagent Kit (TaKaRa, Dalian, China) according to the manufacturer’s instructions. Subsequently, quantitative real time-PCR (RT-PCR) was performed using the CFX96 Real-time PCR detection system (Bio-Rad, United States). The primers that were used in this study are shown in Table 1. The reaction procedure was as follows: 95 ℃, 2 min; 95 ℃, 5 s, 40 cycles; 60 ℃, 30 s; 95 ℃, 5 s; 60 ℃, 5 s; 95 ℃, 2 min. Relative gene expression levels were normalized against the expression levels of β-actin.

**Table 1:** **Sequences of the primers and probes used for qPCR.**

Target genes		Primer sequences (5′- 3′)
β-actin	Forward	GTG ACG TTG ACA TCC GTA AAG A
	Reverse	GCC GGA CTC ATC GTA CTC C
IL-1β	Forward	GAA ATG CCA CCT TTT GAC AGT G
	Reverse	TGG ATG CTC TCA TCA GGA CAG
NLRP3	Forward	ATT ACC CGC CCG AGA AAG G
	Reverse	CAT GAG TGT GGC TAG ATC CAA G
CXCL-1	Forward	ACT GCA CCC AAA CCG AAG TC
	Reverse	TGG GGA CAC CTT TTA GCA TCT T
CXCL-2	Forward	CCA ACC ACC AGG CTA CAG G
	Reverse	GCG TCA CAC TCA AGC TCT G
IL-6	Forward	TCC AGT TGC CTT CTT GGG AC
	Reverse	GTG TAA TTA AGC CTC CGA CTT G
IL-12	Forward	GTC CTC AGA AGC TAA CCA TCT CC
	Reverse	CCA GAG CCT ATG ACT CCA TGT C
TNF-α	Forward	CTC CAG CTG GAA GAC TCC TCC CAG
	Reverse	CCC GAC TAC GTG CTC CTC ACC
RACK1	Forward	AGG GCC ACA ATG GAT GGG TA
	Reverse	CTG GTC AGC TTC CAC ATG ATG

### Coimmunoprecipitation analysis

Cells were prepared and infected with PmCQ2 as described above. After infection, the cells were washed with ice-cold PBS and lysed in ice-cold cell lysis buffer (20 mM Tris at pH 7.5, 150 mM NaCl, 1% Triton X-100, and sodium pyrophosphate, β-glycerophosphate, EDTA, Na_3_VO_4_, and leupeptin) (Beyotime, Beijing, China) for Western blotting and immunoprecipitation (IP). Cell lysates were centrifuged at 12 000 rpm for 10 min at 4 ℃ and then incubated with the indicated antibodies and control IgG antibody overnight at 4 ℃. Then, target proteins that were bound to the antibodies were pulled down with protein A + G beads (Beyotime, Beijing, China) and subjected to immunoblotting analysis.

### SiRNA interference

Cells were prepared as described above and transfected with 60 nM RACK1 siRNA (Sangon Biotech, 5′-CCACAAUGGAUGGGUAACACATT − 3′) or 60 nM control siRNA (Sangon Biotech, 5′-UUCUCCGAACGUGUCACGUTT-3′) using Lipofectamine 3000 (Thermo Fisher Scientific, USA) for 48 h. Then, cells were infected with PmCQ2 as previously described. Subsequently, cell supernatants and lysates were collected for ELISA, Western blotting and RT‒PCR analysis.

### Immunofluorescence staining

To investigate the colocalization of RACK1, NLRP3 and NEK7, cells were prepared as described above and transfected with the pcDNA3.1-eGFP-NEK7 plasmid for 48 h. Then, the cells were infected with PmCQ2 for 3 or 4 h. After infection, the cells were washed three times with PBS and fixed in 4% paraformaldehyde (Sango Biotech, Shanghai, China) for 30 min at room temperature (RT). After three wash steps, the cells were permeabilized with 0.1% Triton X-100 in PBS for 10 min and then blocked with 5% bovine serum albumin (BSA) in PBS for 1 h at RT. After three wash steps, the cells were stained with primary antibodies including anti-NLRP3 (Bioss, Beijing, China), anti-ASC (Santa Cruz, CA, USA) and CoraLite®594-conjugated RACK1 (Proteintech, China) at 4 ℃ overnight. Next, secondary Abs including goat anti-mouse IgG (H&L) Alexa Fluor 488 (Abcam, UK), goat anti-rabbit IgG (H&L) Alexa Fluor 594 (Abcam, UK) and ABflo™ 647-conjugated goat anti-rabbit IgG (H&L) (Abclonal, China) were added after washing with PBS and incubated for 1 h at RT. Subsequently, DAPI (Beyotime Biotechnology, Shanghai, China) was added and incubated in the dark for 5 min. Finally, anti-fluorescence attenuation mounting tablets (Solarbio, Beijing, China) were used, and the results were observed using fluorescence microscopy (Olympus, Tokyo, Japan).

### Statistical analysis

All the data are presented as the mean ± SEM of three independent experiments for each group (*n* = 3). One-way ANOVA was used to analyse the statistical significance of differences among the groups. All the graphs were generated using GraphPad Prism software (San Diego, CA, USA). Statistical significance is shown as the p value, *p < 0.05, **p < 0.01, ***p < 0.001, ns = no significance.

## Results

### *Pasteurella multocida* infection inhibits RACK1 expression

**Figure 1:**
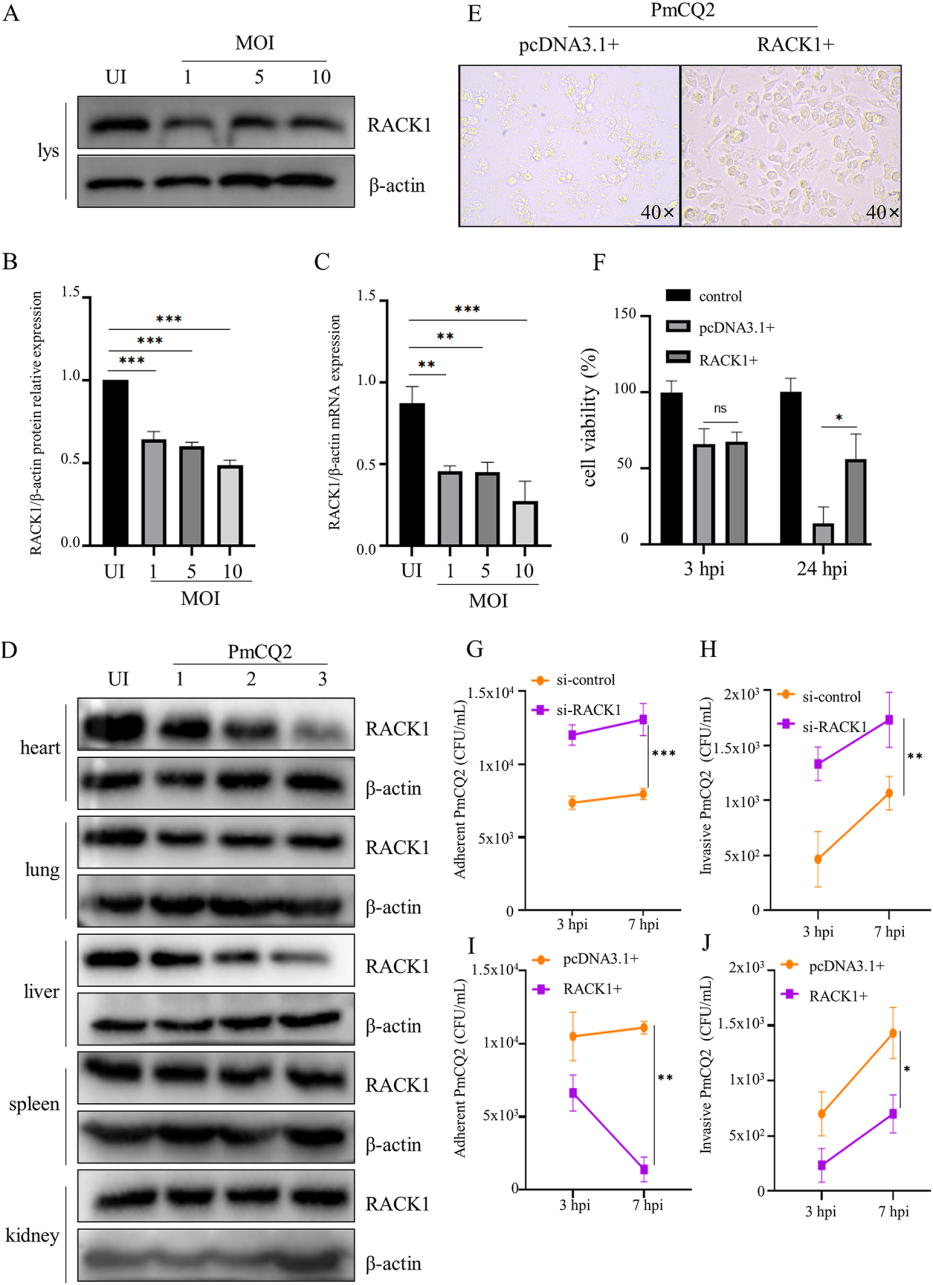
***P. multocida* infection inhibits RACK1 expression. A** Peritoneal macrophages from C57BL/6 mice were infected with PmCQ2 at MOIs of 1, 5, and 10 for 9 h. Subsequently, ciprofloxacin (100 µg/mL) was added, and the cells were cultured for 15 h. Then, cell lysates were collected to measure RACK1 protein expression by immunoblotting. **B** ImageJ was used to quantify the ratio of RACK1 expression to β-actin expression. **C** RACK1 mRNA expression was analysed by RT‒PCR. **D** C57BL/6 (*n* = 3) mice were intranasally infected with PmCQ2 (1000 CFU), and sterilized PBS was used as a control. At 48 h post-infection (hpi), the heart, lung, liver, spleen and kidney were collected and homogenized to measure RACK1 protein expression. **E** Cell morphology is shown. The cells were transfected with pcDNA3.1 and pcDNA3.1-RACK1 plasmids and then infected with PmCQ2 as described above. **F** The viability of RACK1-overexpressing cells was quantified after PmCQ2 infection by Cell Counting Kit-8 assays. **G–J** The number of adherent and invasive PmCQ2 in si-RACK1 cells (G and H) and RACK1-overexpressing cells (I-J), respectively. The data in B, E are presented as the mean ± SEM from three independent experiments with triplicate samples per experiment. * *P* ≤ 0.05; ** *P* ≤ 0.01; *** *P* ≤ 0.001; ns represents no significance.

It has been reported that the expression of RACK1 could be regulated by infection with different pathogens, such as *M.tb* [21], *Helicobacter pylori* [23], porcine reproductive and respiratory syndrome virus (PRRSV) [24], Zika virus [25] and hepatitis C virus [26]. First, we investigated whether RACK1 protein and mRNA expression levels can be regulated by *P. multocida* infection in primary mouse peritoneal macrophages. As shown in Figures 1A-C, RACK1 protein and mRNA expression levels were significantly decreased by infection with different MOIs of *P. multocida* at 24 h post-infection (hpi). Similarly, the expression levels of RACK1 in heart, lung and liver tissues were significantly decreased at 48 hpi (Figure 1D). Notably, *P. multocida* infection induced cell swelling and rupture, as observed by light microscopy, but overexpression of RACK1 inhibited *P. multocida*-induced cell damage (Figure 1E). Consistent with the pathological changes in cells, cell viability was decreased by *P. multocida* infection at 24 hpi, while overexpression of RACK1 by pcDNA3.1-RACK1 transfection significantly rescued cell viability (Figure 1F). Next, to investigate whether RACK-1-mediated cell viability is associated with the amount of bacterial infection, the numbers of adherent and invasive PmCQ2 bacteria were counted at 3 and 7 hpi, respectively. The results showed that the knockdown of RACK1 resulted in an increase in adherent and invasive PmCQ2 (Figures 1G and H). However, the adhesion and invasion of PmCQ2 was significantly reduced in RACK1-overexpressing cells (Figures 1I and J). These results indicate that RACK1 plays a protective role in the host defence against *P. multocida*.

### RACK1 is essential for ***P. multocida***-induced NLRP3 inflammasome activation

**Figure 2:**
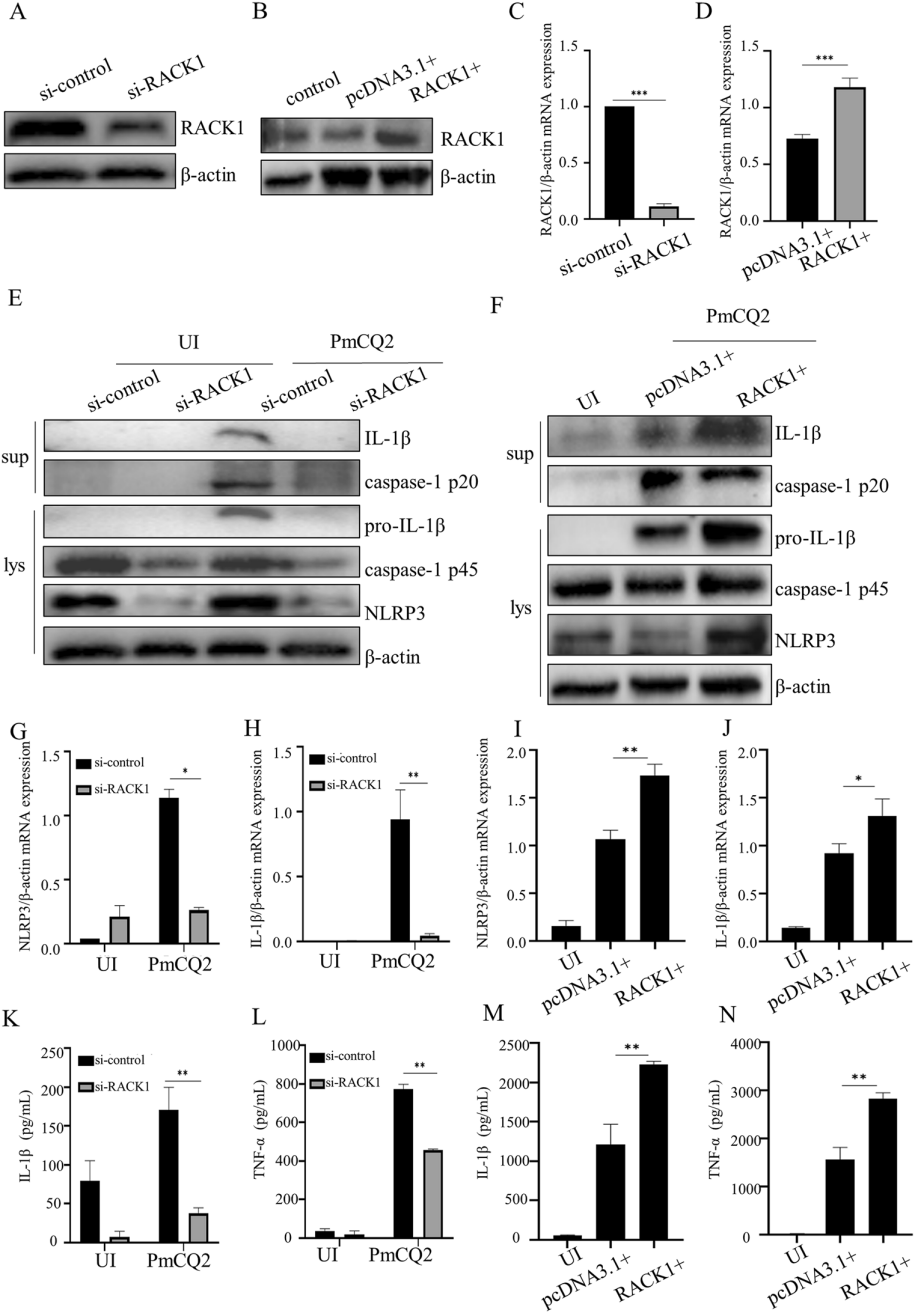
** RACK1 is essential for** ***P. multocida*-induced NLRP3 inflammasome activation.** RACK1 protein expression in RACK1-knockdown macrophages (**A**) and RACK1-overexpressing macrophages (**B**) was measured by Western blotting. RACK1 mRNA expression in RACK1-knockdown macrophages (**C**) and RACK1-overexpressing macrophages (**D**) was measured by RT‒PCR. After 48 h of si-RACK1 and pcDNA3.1-RACK1 plasmid transfection, the cells were infected with PmCQ2 for 9 h, and then ciprofloxacin (100 µg/mL) was added and incubated for an additional 15 h. After 24 h of incubation, supernatants and cell lysates were collected. The protein levels of NLRP3, IL-1β (p31, p17), and caspase-1 (p45, p20) in RACK1-knockdown macrophages (**E**) and RACK1-overexpressing macrophages (**F**) were measured. The NLRP3 and IL-1β mRNA expression levels in si-RACK1 cells (**G**, **H**) and RACK1-overexpressing cells (**I**, **J**) were analysed by RT‒PCR. The levels of IL-1β and TNF-α secreted by si-RACK1 cells (K-L) and RACK1-overexpressing cells (M-N) were measured by ELISA. The data are presented as the mean ± SEM from three independent experiments with triplicate samples per experiment. *P ≤ 0.05; **P ≤ 0.01; ***P ≤ 0.001.

To investigate whether RACK1 mediates PmCQ2-induced NLRP3 inflammasome activation, RACK1 expression was genetically regulated in primary macrophages using siRNA and overexpression technology. The results showed that the transcription and protein expression of RACK1 in macrophages were significantly decreased and increased after siRNA transfection and pcDNA3.1-RACK1 transfection, respectively (Figures 2A–D), demonstrating that we successfully knocked down and overexpressed RACK1 in primary macrophages. Furthermore, knockdown of RACK1 significantly inhibited the secretion of IL-1β and TNF-α (Figures 2K and L) as well as the transcription of NLRP3 and IL-1β (Figures 2G and H) during PmCQ2 infection. Similarly, PmCQ2-induced protein expression of caspase-1, IL-1β and NLRP3 was also significantly attenuated in RACK1-knockdown cells (Figure 2E). Next, to further explore the role of RACK1 in PmCQ2-mediated NLRP3 inflammasome activation, cells were transfected with the pcDNA3.1-RACK1 plasmid and overexpressed RACK1. The results showed that overexpression of RACK1 markedly increased the *P. multocida*-induced secretion of IL-1β and TNF-α (Figures 2M and N) as well as the transcription of NLRP3 and IL-1β (Figures 2I and J). Consistently, caspase-1, IL-1β and NLRP3 protein expression was also significantly increased in RACK1-overexpressing cells (Figure 2F) during *P. multocida* infection. These results demonstrate the involvement of RACK1 in NLRP3 inflammasome activation in response to PmCQ2 infection.

### RACK1 promotes the *P. multocida*-induced formation of ASC specks

**Figure 3:**
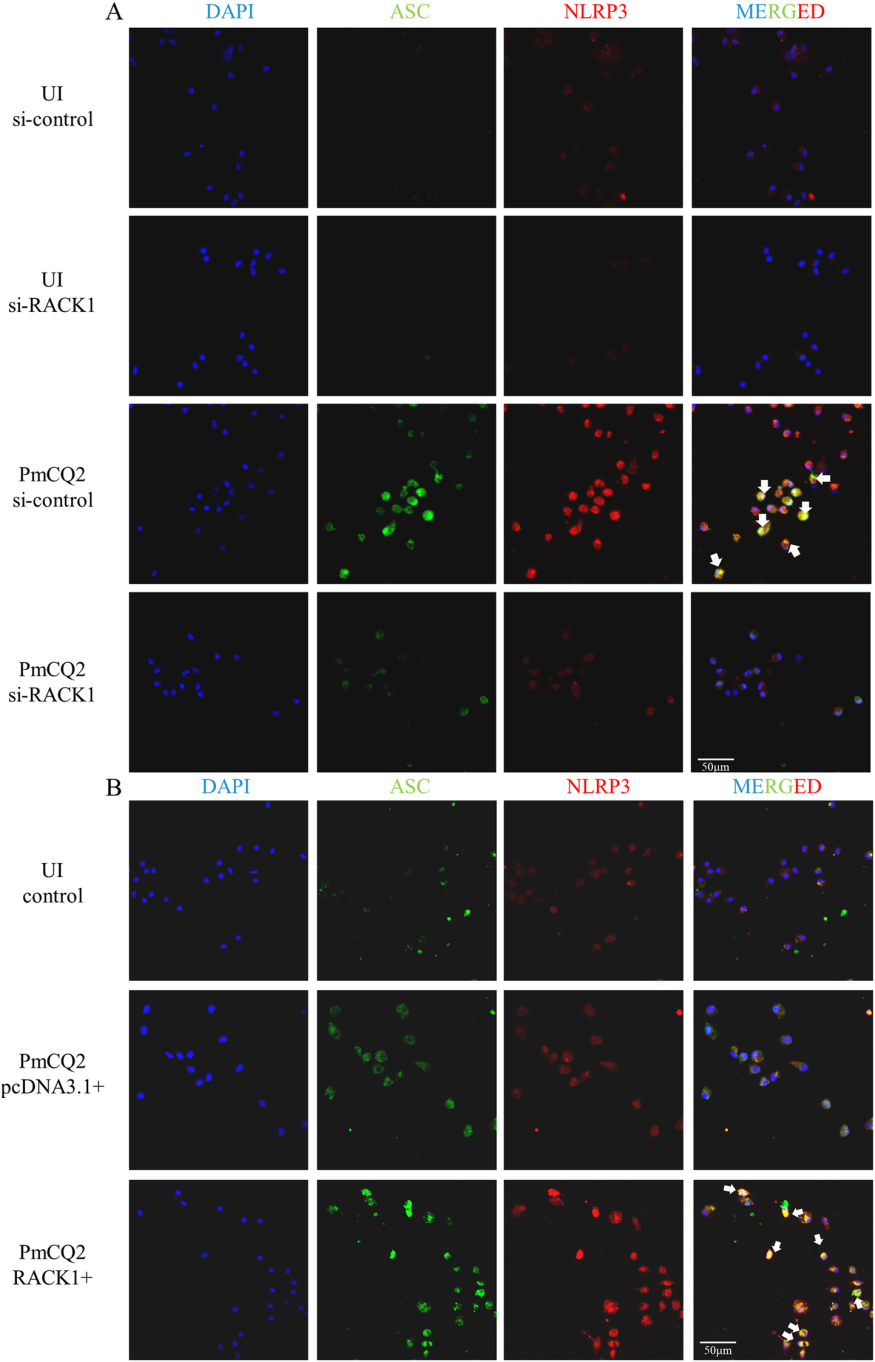
** RACK1 promotes** ***P. multocida*-induced formation of ASC specks.** Cells were transfected with si-RACK1 to knockdown RACK1 and pcDNA3.1-RACK1 plasmid to overexpress RACK1 for 48 h. Then, cells were infected for 24 h as described above. Finally, immunofluorescence staining was performed to detect NLRP3 and ASC specks in RACK1-knockdown cells (**A**) and RACK1-overexpressing cells (**B**). White arrows represent the colocalization of ASC and NLRP3. The images are representative of three independent experiments. The bar in each microscopic image indicates 50 μm.

To further assess the mechanism by which RACK1 mediates PmCQ2-induced NLRP3 inflammasome activation, we investigated the formation of ASC specks, which serve as a platform for the activation of the NLRP3 inflammasome. The immunofluorescence staining results showed that ASC speck formation was induced by *P. multocida* and that these specks colocalized with the NLRP3 protein (Figure 3A, white arrows), but RACK1 knockdown significantly decreased ASC speck formation in macrophages. Moreover, RACK1 overexpression resulted in increased *P. multocida*-induced ASC speck formation and colocalization with the NLRP3 protein (Figure 3B). These results demonstrate that RACK1 mediates *P. multocida*-induced NLRP3 inflammasome activation via the formation of ASC specks.

### RACK1 promotes ***P. multocida***-induced NF-κB activation

**Figure 4:**
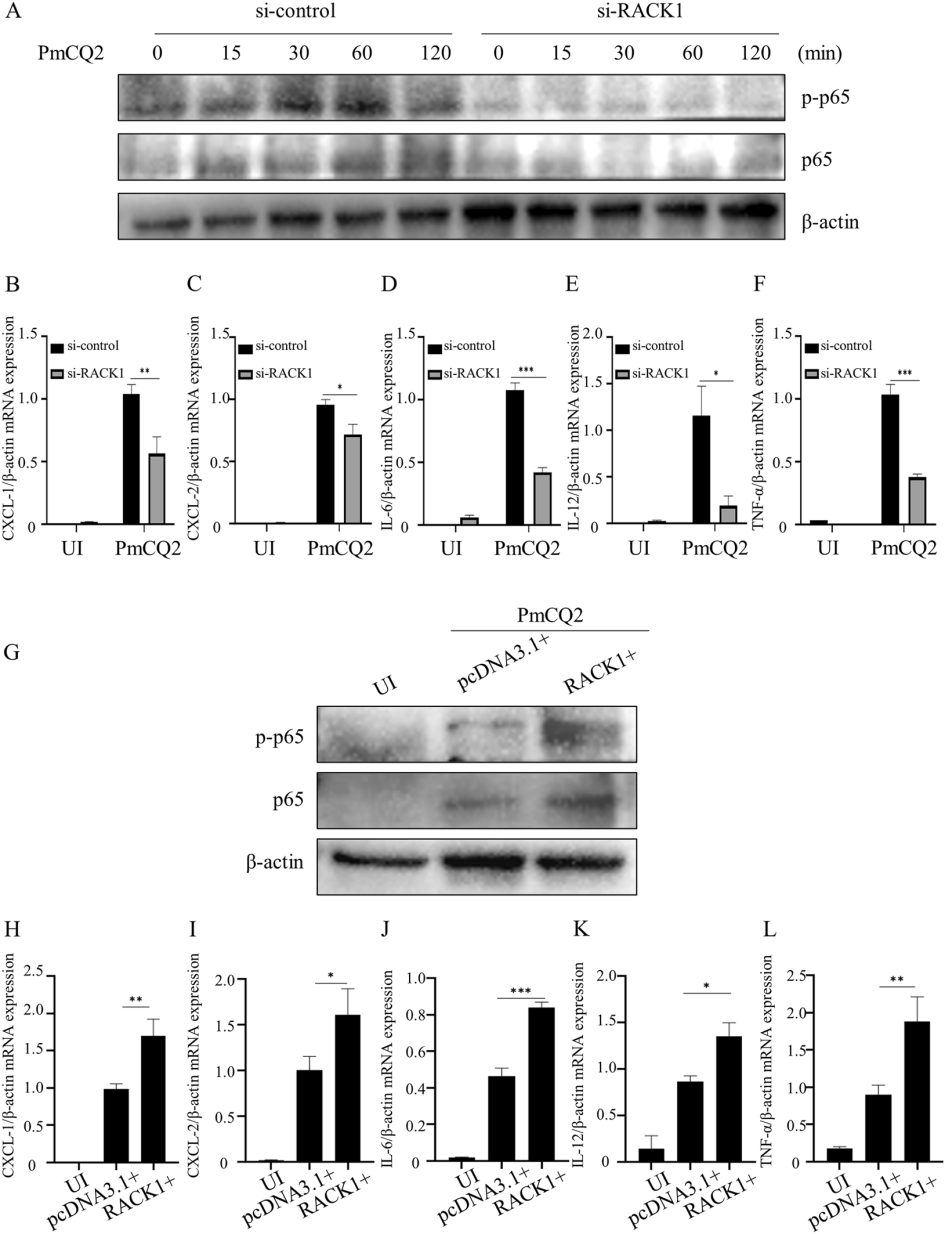
** RACK1 promotes** ***P. multocida*-induced NF-κB activation.** Cells were transfected with si-RACK1 for 48 h and then infected with PmCQ2 for the indicated times. Next, cell lysates were collected, and the protein expression of p65 and p-p65 was measured by Western blotting analysis (**A**). At 6 hpi, the mRNA expression levels of CXCL-1 (**B**), CXCL-2 (**C**), IL-6 (**D**), IL-12 (**E**) and TNF-α (**F**) were measured by RT‒PCR. Similarly, the protein expression of p65 and p-p65 in RACK1-overexpressing cells was also measured by Western blotting analysis. Moreover, the mRNA expression levels of CXCL-1 (**H**), CXCL-2 (**I**), IL-6 (**J**), IL-12 (**K**) and TNF-α (**L**) were measured by RT‒PCR. The data are presented as the mean ± SEM from three independent experiments with triplicate samples per experiment. *P ≤ 0.05; **P ≤ 0.01; ***P ≤ 0.001.

The results showed that knockdown of RACK1 significantly inhibited the *P. multocida*-induced expression of p65 and phosphorylated p65 (Figure 4A) as well as the mRNA expression of CXCL-1, CXCL-2, IL-6, IL-12 and TNF-α (Figures 4B–F) in macrophages. However, RACK1 overexpression significantly increased the *P. multocida*-induced expression of p65 and phosphorylated p65 (Figure 4G) as well as the mRNA expression of CXCL-1, CXCL-2, IL-6, IL-12 and TNF-α (Figures 4H–L) in macrophages.

### *P. multocida* infection promotes the interaction of RACK1 with NLRP3 and NEK7

**Figure 5:**
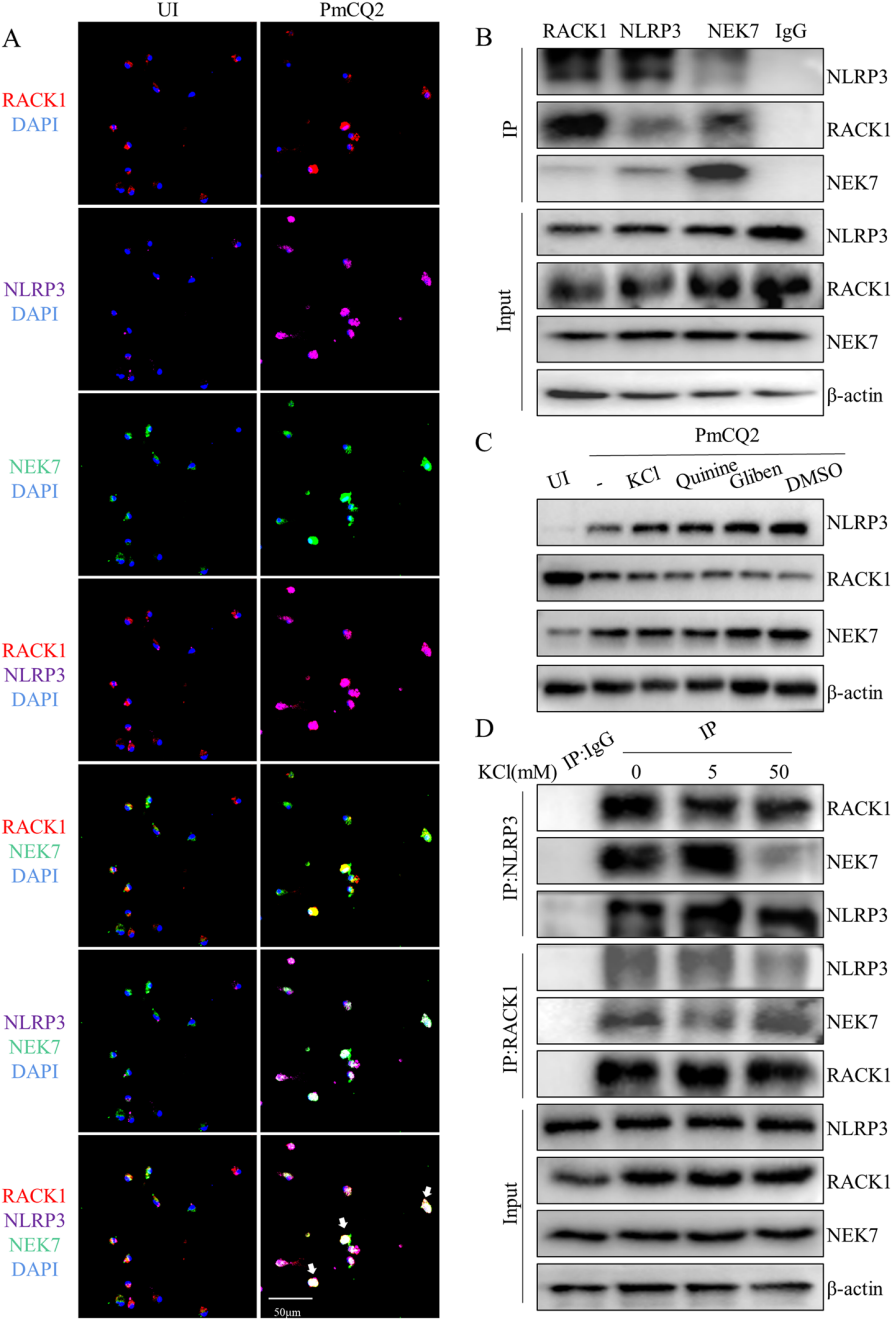
***P. multocida*** **infection promotes the interaction of RACK1 with NLRP3 and NEK7.** Cells were transfected with the pcDNA3.1-eGFP-NEK7 plasmid for 48 h and then infected with PmCQ2 for 24 h. Subsequently, immunofluorescence staining was performed to detect RACK1 and NLRP3 (**A**). The RACK1 protein is shown in red, the NLRP3 protein is shown in purple, the NEK7 protein is shown in green, nuclei are shown in blue. The images are representative of three independent experiments. The bar in each microscopic image indicates 50 μm. Cells were infected with PmCQ2 for 24 h, and then the RACK1-NLRP3-NEK7 interaction was detected by immunoprecipitation and immunoblotting (**B**). Cells were pretreated with KCl, quinine or glibenclamide to inhibit K^+^ efflux and then infected with PmCQ2 for 24 h. RACK1, NLRP3 and NEK7 protein expression (**C**) and the RACK1-NLRP3-NEK7 interaction (**D**) were analysed by Western blotting as well as immunoprecipitation and immunoblotting, respectively. The images and blots are representative of three independent experiments.

Our previous study showed that NEK7 plays an important role in PmCQ2-induced NLRP3 inflammasome activation [12]. The immunofluorescence staining results showed that RACK1, NLRP3 and NEK7 were diffusely distributed in the cytoplasm in uninfected cells, while PmCQ2 infection induced the colocalization of RACK1 with NLRP3 and NEK7 (Figure 5A, white arrows). Furthermore, endogenous coimmunoprecipitation experiments showed that PmCQ2 infection induced the interaction of RACK1 with NLRP3 and NEK7 in macrophages (Figure 5B). To further explore the mechanism underlying the PmCQ2-induced RACK1-NLRP3-NEK7 interaction, inhibitors of K^+^ efflux, including KCl, gliben and quinine, were used. The results showed that the inhibition of K^+^ efflux did not affect the protein expression of RACK1, NLRP3 and NEK7 (Figure 5C) but significantly attenuated the PmCQ2-induced RACK1-NLRP3-NEK7 interaction (Figure 5D). These results demonstrate that K^+^ efflux acts as a critical upstream signal that mediates the RACK1-NLRP3-NEK7 interaction during PmCQ2 infection.

## Discussion

*P. multocida* causes different infectious diseases in animals and humans, and in particular, it causes severe respiratory diseases in bovines that are associated with high mortality rates. Antibiotics are mainly used to treat *P. multocida* infection, but the overuse of antibiotics results in the development of bacterial resistance [27]. In addition, there are no effective vaccines to efficiently prevent *P. multocida* infection, so new strategies are needed to treat or prevent *P. multocida* infection. The innate immune system is the first line of host defence against microbial infection; therefore, research on the pathogenesis of *P. multocida* and its interaction with the host will provide new insights into the identification of novel strategies to control *P. multocida*.

The inflammasome is an important component of the innate immune system, and its activation plays a critical role in the clearance of pathogens via the release of inflammatory cytokines, including IL-1β and IL-18. Our previous study reported changes in gene expression during the host response to the highly virulent *P. multocida* strain PmCQ2, and genomic analysis suggested that the genes that were altered were enriched in the NOD-like receptor signalling pathway [28]. Furthermore, our recent study showed that *P. multocida* induces NLRP3 inflammasome activation, leading to proinflammatory cytokine secretion and an inflammatory response [11, 12, 29]. However, the upstream signalling mechanism underlying PmCQ2-induced NLRP3 inflammasome activation is poorly understood.

It has been reported that RACK1 serves as a scaffold protein for different kinases and receptors, such as PKCβII [30], PRKCE [31], and Flt1 [32], that play essential roles in multiple biological responses. For example, RACK1 inhibits the recruitment of macrophages and regulates the differentiation of macrophages into the M2 macrophages, leading to an anti-inflammatory response, which promotes the development of tumours [18]. Wu et al. found that RACK1 plays a regulatory role in the inflammation of diabetic nephropathy by interacting with the NF-κB subunits p50 and p65 [33]. Brugier et al. found that RACK1 facilitates dengue virus infection by binding to the 40 S ribosomal subunit to recruit Vigilin and SERBP1, which is proposed to link viral RNA to the translation machinery to promote viral replication [34]. In addition, RACK1 expression is significantly upregulated during infection with various pathogens, including *M.tb* [21] and PRRSV [24]. These studies indicate that RACK1 plays a critical role in the host response to infectious and noninfectious diseases. Our study showed that *P. multocida* infection significantly downregulated RACK1 expression in vitro and in vivo. Moreover, RACK1 inhibited the attachment and invasion of PmCQ2 in host cells to reduce infection, indicating that RACK1 serves as a positive regulator of the host defences against *P. multocida* infection, but the detailed mechanism needs to be further studied.

Interestingly, we found that RACK1 knockdown and overexpression significantly abolished and promoted *P. multocida*-induced NLRP3 inflammasome activation, respectively, indicating that RACK1 is essential for *P. multocida*-induced NLRP3 inflammasome activation. Similarly, it has been reported that RACK1 is required for NLRP3 inflammasome activation in LPS + ATP stimulated macrophages. In contrast, RACK1 knockdown has no effect on AIM2 and NLRC4 inflammasome activation that is induced by poly (deoxyadenylic-deoxythymidylic) acid (poly(dA:dT)) and *Salmonella*, respectively [20]. These studies suggest that RACK1 is specifically required for NLRP3 inflammasome activation. It has been reported that RACK1 depletion inhibited NLRP3 inflammasome activation but did not affect the LPS-induced protein expression of NLRP3 and caspase-1 p45 in immortalized bone marrow-derived macrophages (iBMDMs) [20]. In contrast, our study showed that knockdown of RACK1 significantly decreased the protein expression of NLRP3 and caspase-1 p45, as well as the production of pro-inflammatory cytokines. The inhibition of the NLRP3 inflammasome caused by RACK1 knockdown may occur due to the different types of cells that were cultured under different conditions.

Full activation of the NLRP3 inflammasome requires two signals. Bacterial or pathogenic molecules, such as lipoprotein and LPS, are the first signals that activate the NF-κB signalling pathway and induce the transcriptional and post-translational modification of NLRP3. Then, a bacterial toxin or ATP acts as a second signal to activate the NLRP3 inflammasome through the adaptor protein ASC and to recruit caspase-1 [35]. Our study showed that RACK1 mediated *P. multocida*-induced NF-κB activation. Cytokines and chemokines, including CXCL-1, CXCL-2, IL-6, IL-12 and TNF-α, which are dependent on NF-κB activation, were downregulated by knockdown of RACK1, but RACK1 overexpression upregulated the expression of these cytokines and chemokines. In contrast, it has been reported that RACK1 is a negative regulator of NF-κB signalling in TNF-α-treated 293T cells [36], classical swine fever virus-infected PK-15 cells [37] and *Helicobacter pylori*-infected gastric cancer cells [23]. However, RACK1 has been reported to be a positive regulator of NF-κB signalling in high glucose-treated mesangial cells [33] and PRRSV-infected Marc-145 cells [24, 38]. These studies demonstrate that RACK1 is involved in the regulation of NF-κB activation, but it exerts positive or negative regulatory effects in response to different pathogens and different diseases.

Recently, NEK7 was identified as an essential component that regulates the assembly and activation of the NLRP3 inflammasome. It has been reported that the binding of the NEK7 protein to NLRP3 mediates NLRP3 inflammasome activation [39]. Our previous study showed that *P. multocida* infection induces the NEK7-NLRP3 interaction and that NEK7 knockdown significantly inhibits *P. multocida-*induced NLRP3 activation. The present study showed that RACK1 mediated the *P. multocida-*induced NEK7-NLRP3 interaction by binding to NEK7 and NLRP3. In addition, bacterial virulence proteins have been shown to interact with RACK1. For example, RACK1 is reported to bind to the virulence protein YopK from *Yersinia pseudotuberculosis*, which contributes to efficiently avoiding bacterial phagocytosis [40]. Furthermore, it has been reported that macrophage pyroptosis-related proteins from *M.tb* also induce the RACK1-NLRP3 interaction in macrophages, leading to gasdermin D activation, which results in cell apoptosis [21]. These studies suggest that RACK1 is involved in the host defence against pathogens via its direct binding to microorganisms or host factors. However, the mechanism by which the virulence proteins of *P. multocida* promote the RACK1-NLRP3 interaction needs to be further studied.

K^+^ efflux has been considered to be a common trigger of NLRP3 inflammasome activation [35, 41]. Our previous study showed that K^+^ efflux mediates the *P. multocida*-induced NEK7-NLRP3 interaction [12]. The present study showed that RACK1 binds to the NLRP3 and NEK7 proteins to promote NLRP3 inflammasome activation in response to *P. multocida* infection, and this process is mediated by K^+^ efflux. Although RACK1 is involved in inflammasome activation, it is still unclear whether K^+^ channel receptors orchestrate NLRP3 inflammasome activation.

In summary, our study revealed the role of RACK1 in *P. multocida*-induced NLRP3 inflammasome activation. First, RACK1 expression was downregulated by *P. multocida* infection, and RACK1 overexpression prevented *P. multocida-*induced cell death. Furthermore, RACK1 positively regulated *P. multocida-*induced activation of NF-κB and the NLRP3 inflammasome by directly binding to NLRP3. Importantly, this process was mediated by K^+^ efflux. This study demonstrates the important role of RACK1 in controlling inflammation, and RACK1 could be considered a promising therapeutic target for treating microbial infections.
